# Polish experience of lenalidomide in the treatment of lower risk myelodysplastic syndrome with isolated del(5q)

**DOI:** 10.1186/s12885-015-1444-1

**Published:** 2015-07-08

**Authors:** Aleksandra Butrym, Ewa Lech-Maranda, Elżbieta Patkowska, Beata Kumiega, Maria Bieniaszewska, Andrzej Mital, Krzysztof Madry, Tigran Torosian, Ryszard Wichary, Justyna Rybka, Krzysztof Warzocha, Grzegorz Mazur

**Affiliations:** 1 Department of Haematology, Blood Neoplasms and Bone Marrow Transplantation, Wroclaw Medical University, Wroclaw, Poland; 2 Department of Physiology, Wroclaw Medical University, Wroclaw, Poland; 3 Department of Hematology, Institute of Hematology and Transfusion Medicine, Warsaw, Poland; 4 Centre of Postgraduate Medical Education, Warsaw, Institute of Hematology and Transfusion Medicine, Warsaw, Poland; 5 Department of Oncological Hematology, Carpathian Oncology Centre, Brzozow, Poland; 6 Department of Hematology and Transplantology, Medical University of Gdansk, Gdansk, Poland; 7 Department of Hematology, Warsaw Medical University, Warsaw, Poland; 8 Department of Hematology and Bone Marrow Transplantation, Silesian Medical University, Katowice, Poland; 9 Department of Internal Medicine, Occupational Diseases and Hypertension, Wroclaw Medical University, Wroclaw, Poland; 10 Department of Hematology, Institute of Hematology and Transfusion Medicine, Warsaw, Poland

**Keywords:** Myelodysplastic syndrome, del(5q), Lenalidomide, Transfusion independence

## Abstract

**Background:**

Lenalidomide has been approved for the treatment of lower-risk myelodysplastic syndrome (MDS) with 5q deletion (del(5q)). We present for the first time a retrospective analysis of low-risk MDS with isolated del5q treated with lenalidomide, outside the clinical trials.

**Methods:**

36 red blood cell (RBC) transfusion-dependent patients have been included in the study. Patients received lenalidomide 10 mg/day on days 1–21 of 28-day cycles.

**Results:**

91.7 % of patients responded to lenalidomide treatment: 72.2 % achieved erythroid response, 19.4 % achieved minor erythroid response and 8.4 % of patients did not respond to treatment. Response depended on number of previous treatment lines (*p* = 0.0101), International Prognostic System Score (IPSS; *p* = 0.0067) and RBC transfusion frequency (*p* = 0.0139). Median duration of response was 16 months (range 6–60 months). Treatment was well tolerated. We observed hematological toxicity (grade 3 and 4): neutropenia in 16 (44.4 %) patients and thrombocytopenia in 9 (25 %) patients. Two patients (5.5 %) progressed to high-risk MDS and two subsequent progressed to acute myeloid leukemia. A Kaplan-Meier estimate for overall survival at 5 years in the study group was 79.0 ± 8.8 %.

**Conclusions:**

Lenalidomide in this group of patients was beneficial for the treatment of RBC transfusion-dependency with well-known safety profile.

## Background

In 2005, lenalidomide as an immunomodulating agent was approved by U.S. Food and Drug Administration (FDA) in treatment of transfusion-dependent patients with low-risk myelodysplastic syndrome (MDS) with 5q deletion (del(5q)). Currently, chromosome 5q deletion is one of the most frequent rearrangements observed in myelodysplastic syndrome, which may exist as an independent aberration or as a complex of cytogenetic disorders. Based on the latest classification of the World Health Organization (WHO), a new nosological entity, isolated deletion, was described. The above mentioned entity is characterized by the well-known clinical disease manifestation: presence of <5 % of myeloblasts in bone marrow and lack of Auer rods [1, 2]. Patients with isolated 5q deletion are mainly females with macrocytic anemia in the peripheral blood accompanied by normal platelet count or thrombocytosis. In the majority of patients with classic del(5q) syndrome, a transfusion dependency accompanied by iron overload developed over time [3]. In the paper by Patnaik et al., it was revealed that age, transfusion needs at diagnosis and dysgranulopoiesis were independent factors affecting survival reduction in patients with chromosome 5 long arm deletion syndrome [4]. In patients with del(5q) syndrome, lenalidomide introduction has given the chance for long-term responses to be achieved [5–9]. The drug modifies also immunological pathways involved in pathomechanism of the disease [10].

Currently, available data concerning lenalidomide efficacy in patients with myelodysplastic syndrome and del(5q) comes only from randomized studies. Moreover, the above mentioned data concerns a heterogeneous group of patients (del(5q) with additional aberrations); however, no publication on patients with isolated 5q deletion could be found [11]. In this paper, we present a retrospective analysis of homogeneous group of patients with isolated chromosome 5 long arms deletion treated with lenalidomide in Poland during the period 2006–2013 outside clinical trial settings.

## Methods

This analysis included transfusion-dependent patients with low- and intermediate-1 risk myelodysplastic syndrome with isolated del(5q) treated in 14 Polish hematological centers (2006–2013). Patients were receiving a 21-day oral treatment with lenalidomide 10 mg within a 28-day cycle. Patients were analyzed in respect of treatment response, treatment response duration and overall survival (measured in months since del5q syndrome diagnosis). Treatment response was assessed on the basis of International Working Group Criteria [12]. Treatment toxicity was determined in accordance with Common Toxicity Criteria (CTC) scale v.4.0. The study has been performed in accordance with the Declaration of Helsinki and was approved by Wroclaw medical University ethics committee. Informed consent was not obtained for publication of patient data as it is a retrospective analysis and does not compromise anonymity or confidentiality of patients.

### Statistical analysis

Tests results were analyzed with the use of statistical methods. In all groups, means (X), medians (M), range (min-max), standard deviations (SD) as well as lower and upper quartiles (25-75Q) for investigated continuous parameters were calculated. Hypothesis concerning equivalence of means for particular samples was verified with the use a non-parametric Kruskal-Wallis test due to small number of cases in each group. In case of discrete parameters, the prevalence of a given trait in the group was analyzed with the use of χ^2^_df_ test with Yates' correction and appropriate number of degrees of freedom df (df = (m-1)*(n-1), where m – number of lines, n – number of columns); or in case of 2 × 2 tables, when the expected cell value was < 5, the Fisher test was used. Survival curves were prepared with the use of Kaplan-Meier method. *P* value ≤ of 0.05 was statistically significant. Statistical analysis was performed with the use of computer packages of statistical programs EPIINFO version 7.1.1.14 (from 02.07.2013).

## Results

### Patients’ characteristics

The final assessment was performed in 36 patients with isolated del(5q). The patient group was composed of 32 females (88.9 %) and 4 males between 25 and 83 years of age (median 66 years). In 14 patients, IPPS risk score was 0 and in 22 subjects the score was 0.5. According to IPSS-R classification, there were 25 patients with low risk and 11 patients with intermediate risk. WHO adapted Prognostic Scoring System (WPSS) was also analyzed in two time points: at diagnosis there were 9 patients with WPSS 0 (very low risk) and 27 with WPSS 1 (low risk). At the moment of lenalidomide beginning all patients were in low risk category (WPSS 1).

At the commencement of treatment, all patients were transfusion-dependent. Enumeration of blast cell percentage has been done from a bone marrow aspiration and trephine biopsy. 18 subjects were treatment-naive; 6 patients received 1 previous treatment line; 8 subjects received 2 previous treatment lines and 4 patients received 3 treatment lines (erythropoietin, steroids, thalidomide) before lenalidomide therapy was started. Complete patients’ characteristics are summarized in Table 1.

**Table 1:** Baseline patients’ characteristics

Characteristic	Total number of patients (N = 36)
Age, years	
Median	66
Range	25–83
Sex, n (%)	
Female	32 (88.9)
Male	4 (11.1)
Time since del5q diagnosis (months)	
Median	10
Range	1–83
IPSS risk score, n (%)	
0	14 (38.9)
0.5	22 (61.1)
Number of previous treatment lines	
0	18
1	6
2	8
3	4
Transfusion dependency (months)	
Median	10
Range	2–47
RBC transfusion units/8 weeks	
Median	2
Range	1–10
Absolute neutrophil count (ANC), x 10^9^/l	
Median	1.6
Range	0.4–5.2
Baseline hemoglobin level, g/dl	
Median	7.4
Range	5.2–8
Baseline platelet count, x 10^9^/l	
Median	259
Range	102–742
Myelogram	
High cellularity	30
Low cellularity	6
Bone marrow dysplasia	
1 line	8
2 lines	16
3 lines	12
Lactate dehydrogenase (LDH) level, n (%)	
Normal	13 (36.1)
Increased	23 (63.9)
Ferritin level	
Elevated	26
Missing	10

### Lenalidomide treatment

Patients received 1–20 cycles of lenalidomide therapy (median 5 cycles). 12 patients received ≤3 cycles of therapy and 24 subjects received >3 cycles of treatment. In 7 patients lenalidomide dose was reduced post 2nd and 3rd treatment cycle (in 1 patient due to infection and in 6 persons due to hematological toxicity). In those patients lenalidomide was administered in reduced dose of 5 mg on days 1–28 for two cycles. Then original dose of 10 mg was restarted. In 15 subjects treatment was stopped, despite good response, due to lack of further therapy reimbursement by the National Health Fund. In 9 patients, treatment was completed post erythroid response achievement. Only in 1 patient, lack of response was the cause for treatment withdrawal; in 2 patients, treatment was ended due to response loss (treatment until progression). In 3 patients, treatment was stopped due to toxicity. 6 patients still remain on lenalidomide therapy.

### Treatment response

In 33 (91.7 %) patients, following treatment response was observed: 26 (72.2 %) patients achieved erythroid response; 7 patients obtained minor erythroid response. In 3 patients, no efficacy of lenalidomide therapy was seen. In case of cytogenetic response, a post-treatment follow-up cytogenetic test was performed only in 2 patients. Those patients achieved a complete cytogenetic response. Treatment response achievement (erythroid, cytogenetic) was negatively associated with number of previous treatment lines before lenalidomide administration; *p* = 0.0101 (the number of therapies before lenalidomide did not influence response duration but achievement of response), IPSS risk score (*p* = 0.0067), IPSS-R risk score (*p* = 0.01) and transfusion requirements (patients requiring less transfusions had better treatment response; *p* = 0.0139). Similarly, higher ferritin level was associated with worse treatment response (*p* = 0.0355). Lenalidomide dose reduction during treatment was associated with worse treatment response (*p* = 0.0334). The time from disease initial diagnosis did not affect treatment outcome. In total, in 33 patients transfusion independence was achieved. In 2 other subjects, an interval between two subsequent transfusions was extended. In 17 patients, transfusion independence was already observed after 1 treatment cycle; in 11 subjects after 2 cycles; in 3 after 3 cycles of lenalidomide therapy and in 2 other subjects post 4 treatment cycles. Transfusion independence achievement was associated with the number of previous treatment lines (*p* = 0.0308) but not linked to other clinical features as hemoglobin, platelets, neutrophils, blast count. Post-treatment median hemoglobin level increase was 4 g/dl (range 1–9.1 g/dl). Median duration of treatment response was 16 months (range 6–60 months). In two patients (5.5 %), a progression into high-risk MDS and in two other into acute myeloid leukemia were observed. Patients who progressed to high-risk MDS had no additional changes in cytogenetic. Patients, who progressed to AML had evolution of karyotype: trisomy of chromosome 8 in one patient and complex kariotype in second patient.

### Toxicity

Treatment was well-tolerated. A hematological toxicity (grade 3 and 4) was observed: neutropenia - in 16 (44.4 %) patients and thrombocytopenia in 9 (25 %) subjects. Older age was a factor predisposing to grade 3 and 4 thrombocytopenia occurrence (*p* = 0.0156). Median age in group of patients without thrombocytopenia was 61 while in the group with thrombocytopenia during lenalidomide treatment was 71. In 4 patients, treatment was complicated by pneumonia (grade 2); in two patients, a diarrhea (grade 2) occurred. Other rare complications (grade 1 and grade 2) of therapy were as follows: liver enzymes increase (2 patients) and skin rash (1 patient).

### Survival

**Fig. 1:**
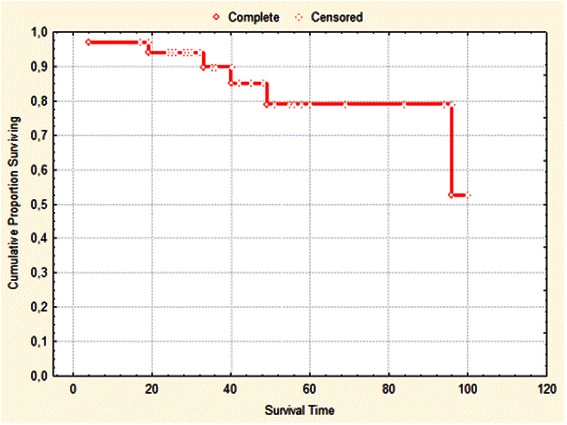
Kapplan-Meier estimate for patients' overall survival proportion

In the study group, a Kaplan-Meier estimate for overall survival at 5 years was 79.0 ± 8.8 % Fig. 1. In multivariate analysis ferritin level was the only independent prognostic factor for longer OS in lenalidomide treated population (*p* = 0.01). In study population, five deaths were seen: one due to infection (during therapy), two deaths due to progression into AML (Acute myeloid leukemia), one due to progression into high-risk MDS and 2 due to unknown reasons. In those patients lenalidomide was stopped before death. Median leukemia free survival was 30 months (range 3–92). Patients' median follow-up was 58 months.

## Discussion

To the best of our knowledge, this paper presents the first analysis of cytogenetically homogeneous group of patients with myelodysplastic syndrome and isolated 5q deletion. It should be highlighted that this study presents results of drug experience used in everyday clinical practice, outside clinical trial settings. In paper from 2005, it was demonstrated that lenalidomide had a hematological activity in patients with low-risk MDS without response to erythropoietin therapy whereas patients with del(5q) have higher response rate (83 %) in comparison to subjects with normal karyotype (57 %) and other chromosomal aberrations (12 %) [13]. In this paper, response rate was higher (91.7 %) due to homogeneous group of patients. In their later work, List et al. demonstrated the high efficacy of lenalidomide in patients with low- and intermediate-1 risk MDS and del(5q) syndrome. In 76 % of examined patients, transfusion need decrease was observed and in 67 % of subjects transfusion independence was achieved [9]. In comparison, our results show an even better response rate (91.7 %). Cytogenetic treatment response was achieved in 73 % of patients. Approximately 90 % of patients achieved transfusion independence within 3 months of therapy (range 1–49 weeks, median 4.6 weeks) [9]. In comparison, results of our analysis demonstrate that 93.9 % of patients achieved transfusion independence within first three treatment cycles. In the study mentioned above, a multivariate analysis showed that response to lenalidomide therapy was negatively affected by the presence of thrombocytopenia and increased transfusion requirements. Similarly, our results indicate that previous transfusion requirement negatively impacts the achievement of response to lenalidomide therapy.

In a phase III randomized trial, MDS-004, efficacy of two lenalidomide doses (5 and 10 mg) in transfusion-dependent patients with low- and intermediate-1 risk level was compared [8]. Transfusion independence for ≥26 weeks was demonstrated in: 56 % of patients treated with lenalidomide 10 mg, in 41 % of subjects receiving lenalidomide 5 mg and in 6 % of patients treated with placebo (*p* < 0.001 in 10 and 5 mg groups vs. placebo). In 10 mg lenalidomide group, 24 % of patients achieved complete cytogenetic response whereas in 5 mg group 11 % of subjects (placebo group-0 %). Adverse events in form of grade 3–4 neutropenia were observed in 74 and 75 % patients according to lenalidomide dose level (15 % in placebo group) whereas thrombocytopenia was seen in 33 and 41 % patients receiving 10 and 5 mg of lenalidomide, respectively (2 % in placebo group). In analysis of patients achieving transfusion-free period >26 weeks, a longer period free of transformation into acute myeloid leukemia in comparison to patients without treatment response was revealed (*p* = 0.021). Factors affecting survival free of progression into AML and overall survival before therapy were as follows: higher ferritin level, older age and high number of transfusions. Bone marrow myeloblast percentage, cytopenias, cytological abnormalities and IPSS risk score were not correlated with the disease progression risk. In the analysed population, the size of group with disease transformation (into AML or higher-risk MDS) is too small for reliable statistical analyses conduction. However, the obtained percentage results are not significantly different from literature data concerning population without lenalidomide treatment [14]. In the study by Le Bras et al., a 48-week lenalidomide therapy in 95 patients with low- and intermediate-1 risk MDS and del(5q) syndrome was described [15]. In the above mentioned study, 63 % of patients achieved transfusion independence. Median time to transfusion independence achievement was 16 weeks whereas mean overall survival after 16 months of therapy was 86 %. Within first 8 weeks of therapy, grade 3–4 neutropenia occurred in 62 % of patients whereas grade 3-4 thrombocytopenia in 25 % of subjects resulting in drug dose reduction in 55 % of cases. In our study, grade 3-4 neutropenia rate was 44 %, thus it was lower in comparison with cited publication. In contrast, thrombocytopenia rate is similar to that observed in Le Bras et al. study.

The study performed by Göhring et al. revealed that patients without cytogenetic remission and erythroid response post lenalidomide therapy had greater risk of progression into acute leukemia [16]. After three and five years of post-enrolment observation, the cumulative percentage of AML occurrence in patients with cytogenetic response was 10 and 21 %, respectively. In contrast, in patients without treatment response those rates were respectively 46 and 60 %. In 37 % of patients with MDS and isolated del5q and normal bone marrow blasts count, a progression into AML was observed whereas in 87 % of patients, a cytogenetic clonal evolution was seen. The weakness of our study group was the absence of cytogenetic analysis in patients with treatment response. The main reason for this seems to be the fact that physicians were satisfied by observing the treatment outcome and interrupted therapy when erythroid response was achieved. It is very important due to results of the last work by List et al., in which authors demonstrated that transfusion independence and cytogenetic response post lenalidomide therapy were associated with longer overall survival and lower progression into AML risk [17].

## Conclusion

According to our knowledge, this is the first efficacy analysis of lenalidomide used outside clinical trial settings in patients with myelodysplastic syndrome with isolated 5q deletion. Currently available data indicates that lenalidomide 10 mg remains the first-line choice in treatment of patients with myelodysplastic syndrome and del(5q) aberration and results in higher transfusion independence and cytogenetic response rates achievement. It is worth noting that patients should be treated until disease progression. In our analysis, the main emerging problem was the limited drug availability resulting from socio-economic conditions, which in turn translated into too short treatment. Despite small numbers of therapeutic cycles, a very high rate of long-term responses (transfusion independence) was achieved. Treatment continuation at the moment of erythroid response achievement increases the chance for cytogenetic response occurrence and thus translated into patients' survival improvement. In the context, socio-economic conditions should play a secondary role and therapy should continue until patient still gets benefits from it.

## Abbreviations

AML: Acute myeloid leukemia
CR: Complete remission
CTC: Common toxicity criteria
FDA: Food and drug administration
IPSS: International prognostic scoring system
IPSS-R: International prognostic scoring system
MDS: Myelodysplastic syndrome
RBC: Red blood cell
SD: Standard deviation
WHO: World health organisation
WPSS: WHO classification-based Prognostic Scoring System
